# Deficits in higher visual area representations in a mouse model of Angelman syndrome

**DOI:** 10.1186/s11689-020-09329-y

**Published:** 2020-10-19

**Authors:** Leah B. Townsend, Kelly A. Jones, Christopher R. Dorsett, Benjamin D. Philpot, Spencer L. Smith

**Affiliations:** 1 Neuroscience Curriculum, University of North Carolina School of Medicine, Chapel Hill, NC 27599 USA; 2 Department of Cell Biology and Physiology, University of North Carolina School of Medicine, Chapel Hill, NC 27599 USA; 3 Carolina Institute for Developmental Disabilities, University of North Carolina School of Medicine, Chapel Hill, NC 27599 USA; 4 Neuroscience Center, University of North Carolina School of Medicine, Chapel Hill, NC 27599 USA; 5 Department of Electrical & Computer Engineering, Neuroscience Research Institute, Center for BioEngineering, University of California Santa Barbara, 2002 BioEngineering Building; Mail code 5100, Santa Barbara, CA 93106-5100 USA

## Abstract

**Background:**

Sensory processing deficits are common in individuals with neurodevelopmental disorders. One hypothesis is that deficits may be more detectable in downstream, “higher” sensory areas. A mouse model of Angelman syndrome (AS), which lacks expression of the maternally inherited *Ube3a* allele, has deficits in synaptic function and experience-dependent plasticity in the primary visual cortex. Thus, we hypothesized that AS model mice have deficits in visually driven neuronal responsiveness in downstream higher visual areas (HVAs).

**Methods:**

Here, we used intrinsic signal optical imaging and two-photon calcium imaging to map visually evoked neuronal activity in the primary visual cortex and HVAs in response to an array of stimuli.

**Results:**

We found a highly specific deficit in HVAs. Drifting gratings that changed speed caused a strong response in HVAs in wildtype mice, but this was not observed in littermate AS model mice. Further investigation with two-photon calcium imaging revealed the effect to be largely driven by aberrant responses of inhibitory interneurons, suggesting a cellular basis for higher level, stimulus-selective cortical dysfunction in AS.

**Conclusion:**

Assaying downstream, or “higher” circuitry may provide a more sensitive measure for circuit dysfunction in mouse models of neurodevelopmental disorders.

**Trial registration:**

Not applicable.

## Introduction

Copy number variants in the *UBE3A* gene, which encodes an E3 ubiquitin ligase, result in neurodevelopmental disorders in humans. Loss of *UBE3A* in neurons underlies Angelman syndrome (AS) [1, 2], and increased *UBE3A* gene dosage via duplications is associated with autism [3–5]. AS is characterized by developmental delays, speech impairments, movement and balance disorders, seizures, and an apparent happy demeanor [6]. Sensory processing abnormalities are also common in individuals affected with AS, including hyper- and hypo-responsivity to visual stimuli [7–9]. Because the paternal allele of *UBE3A* is epigenetically silenced in most neurons [10–14], deficient expression or function of the maternally inherited *UBE3A* allele alone is detrimental and causes AS [1, 15]. Mouse models of AS, which lack maternal *Ube3a* expression, recapitulate many of the phenotypes of AS including seizures, movement and balance deficits, and learning deficits [16–18].

In humans, loss of maternal *UBE3A* produces symptoms that emerge during development, such that individuals with AS often go undiagnosed until 6–12 months of age [6]. The developmental emergence of behavioral phenotypes is also observed in mouse models of AS [17, 19], and coincides with the emergence of specific circuit deficits, while many other circuit functions appear to remain intact. For example, loss of maternal *Ube3a* does not alter retinotopic map formation in primary visual cortex (V1), eye-opening, or acuity development; but it does alter specific V1 microcircuits and impairs ocular dominance plasticity, a measure of the brain’s malleability to adapt to changes in the pattern of visual inputs [20–23]. These findings suggest that functional deficits in processing likely emerge with the developmental refinement of circuitry, but details of these deficits remain unknown.

To determine the effects of maternal *Ube3a* loss on functional processing in the cortex, we examined visually evoked responses in visual cortical areas of both wildtype (WT) mice and a mouse model of AS, which lacks the maternal *Ube3a* allele [18]. Mouse visual cortex consists of V1 and higher visual areas (HVAs) that process visual information [24–28]. Neurons in V1 and HVAs exhibit tuning for visual features, such as orientation, spatial frequency, and temporal frequency [24, 27, 29]. Mice can perform visually guided behavioral tasks that rely on processing complex visual stimuli [30–34]. Thus, the mouse model offers an experimentally accessible transgenic system to investigate the underlying cause of deficits in higher-order visual processing.

We first measured the responsiveness of V1 and HVAs to visual stimuli with intrinsic signal optical imaging (ISOI) at multiple postnatal time points after eye-opening. This revealed that a lack of maternal *Ube3a* produces stimulus-specific deficits in HVA activity and that these deficits develop after an important developmental window: the canonical critical period for ocular dominance plasticity (postnatal days 19–32; P19–P32) [35]. To determine the cellular basis of these neural response deficits, we investigated HVA circuitry at the single neuron level using cell type-specific two-photon population calcium imaging in vivo. Analysis of stimulus-evoked neuronal activity modulation in excitatory and inhibitory neurons revealed that the stimulus-specific deficit was linked to a decrease in inhibitory interneuron activity. Our findings reveal the functional consequences of maternal *Ube3a* loss on higher cortical circuitry and implicate inhibitory interneurons to stimulus-selective circuit deficits.

## Results

**Fig. 1:**
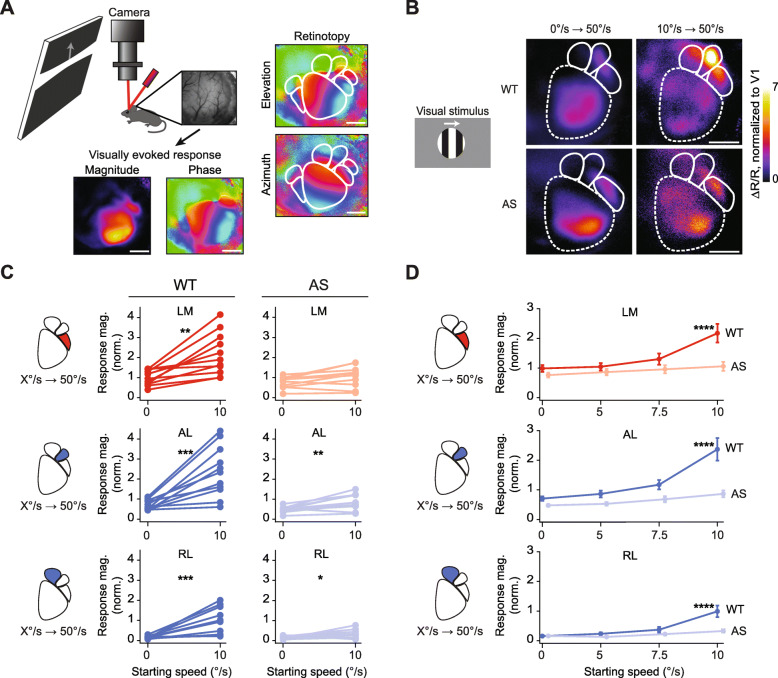
Higher visual areas in Angelman syndrome model mice show stimulus-specific deficits in activity modulation. **a** Schematic of intrinsic signal optical imaging (ISOI) used to identify the boundaries and measure activity in HVAs. Visual stimuli were presented to the contralateral (left) eye to a lightly anesthetized mouse via an LCD screen. Magnitude and phase maps in response to multiple stimuli were obtained. Phase maps evoked by a single drifting bar stimulus were used to determine retinotopy. **b** Representative magnitude maps of primary and higher visual areas of WT and AS model mice in response to still-fast (0➔50°/s) and slow-fast (10➔50°/s) gratings. ΔR/R, normalized to V1. White lines designate boundaries between visual areas determined by retinotopy; solid lines indicate regions quantified in (**c**); dashed lines indicate regions not quantified. Scale bar, 1 mm. **c** Quantification of the amplitude of responses of HVAs LM, AL, and RL to still-fast and slow-fast gratings, normalized to V1 activation for each stimulus. Each animal’s response to both stimuli is connected by a line. Two-tailed paired *t* test. *p < 0.05; **p < 0.01; ***p < 0.001. **d** Stimulus-response curves in HVAs LM, AL, and RL to stimuli in which only the starting speed is changed. LM: stimulus effect (*p* = 0.0001), genotype effect (*p* = 0.0002), and interaction effect (*p* = 0.0211). AL: stimulus effect (p < 0.0001), Genotype effect (p < 0.0001), and interaction effect (*p* = 0.007). RL: stimulus effect (*p* < 0.0001), genotype effect (*p* = 0.0003), and interaction effect (*p* = 0.0008). Two-way ANOVA. Tukey’s post hoc. ****p < 0.0001

We used in vivo ISOI to first map V1 and HVAs simultaneously, and then to measure cortical responses to visual stimuli (the imaging field-of-view was 4.7 mm × 4.7 mm, encompassing V1 and HVAs). For mapping, a single high-contrast drifting bar oriented either horizontally (elevation) or vertically (azimuth) to map retinotopy and locate V1 and HVAs in both AS mice and wildtype (WT) littermates [27, 28, 36–39] (Fig. 1a). Mice of both sexes were used, age 2–4 months. Visually evoked signals from the lateromedial area (LM), anterolateral area (AL), and rostrolateral area (RL) were more reliably detected (Fig. 1b, Supp. 1) than signals from the anteromedial area (AM), posteromedial area (PM), and laterointermediate area (LI) in WT mice. Thus, we focused our subsequent analyses on areas LM, AL, and RL. To control for mouse-to-mouse variability, we normalized HVA response magnitude measurements to that of V1 for each stimulus (Fig. 1, Supp. 2).

### A stimulus-specific deficit of visual responses in HVAs in AS mice

To compare visual responses of AS mice to WT littermates, we first used three stimuli: still-fast gratings, slow-fast gratings, and random-dot kinematograms (RDK). The still-fast grating stimulus consisted of black and white vertical gratings that periodically cycled in drift speed from 0°/s to 50°/s [24, 27]. The slow-fast grating stimulus was identical to the still-fast grating except that it periodically cycled in drift speed from 10°/s to 50°/s [40]. RDK consisted of a field of white dots on a black background, which moved in random directions with 0% coherence [41, 42]. We found that visually evoked responses measured with ISOI were mostly similar in AS and WT mice in response to two of our stimuli. Still-fast gratings produced comparable responses in AS and WT mice in LM and RL (LM: *t*_(20)_ = 1.577, *p* = 0.131; RL: *t*_(20)_ = 0.1471, *p* = 0.885; WT: *N* = 11, AS: *N* = 11) and a detectable difference in AL (AS mice = 0.477 ± 0.044, *N* = 11 vs. WT mice = 0.705 ± 0.072, *N* = 11;, *t*_(20)_ = 2.679, *p* = 0.0144) (Fig. 1b, c). Similarly, in response to RDK, responses were mostly comparable between the two groups except for a small difference found in AL between AS mice (0.266 ± 0.047) and WT mice (0.397 ± 0.033), *t*_(18)_ = 2.327, *p* = 0.032, (WT: *N* = 11, AS: *N* = 9) **(**Supp. 3). Together, our findings revealed that HVAs are largely unaffected in AS mice.

However, in response to the slow-fast grating stimulus, we observed genotypic differences in activity in all HVAs examined. The stimulus produced significantly (*p* < 0.005) stronger HVA responses in WT mice compared to those in AS mice (two-tailed *t* tests; LM: 217 ± 31% in WT, 106 ± 15% in AS, *p* = 0.0047; AL: 236% ± 38% in WT, 86.5 ± 11% in AS, *p* = 0.0012, and RL: 98.9% ± 19% in WT, 32.7 ± 6.1% in AS, *p* = 0.004) (Fig. 1b, c). We compared responses to the two grating stimuli within individual regions and found that response modulations were higher in response to slow-fast gratings than to still-fast gratings in HVAs of WT mice (LM: *t*_(20)_ = 3.546, *p* = 0.002; AL: *t*_(20)_ = 4.296, *p* = 0.0004; RL: *t*_(20)_ = 4.625, *p* = 0.0004) (Fig. 1c). By contrast, in AS mice, we found a much smaller increase in response modulation in HVAs between the two stimuli (LM: *t*_(20)_ = 1.712, *p* = 0.1023; AL: *t*_(20)_ = 3.107, *p* = 0.0056; RL: *t*_(20)_ = 2.529, *p* = 0.0199) (Fig. 1c). Together, these results reveal a stimulus-specific deficit in neuronal responses in HVAs in AS mice.

**Fig. 2:**
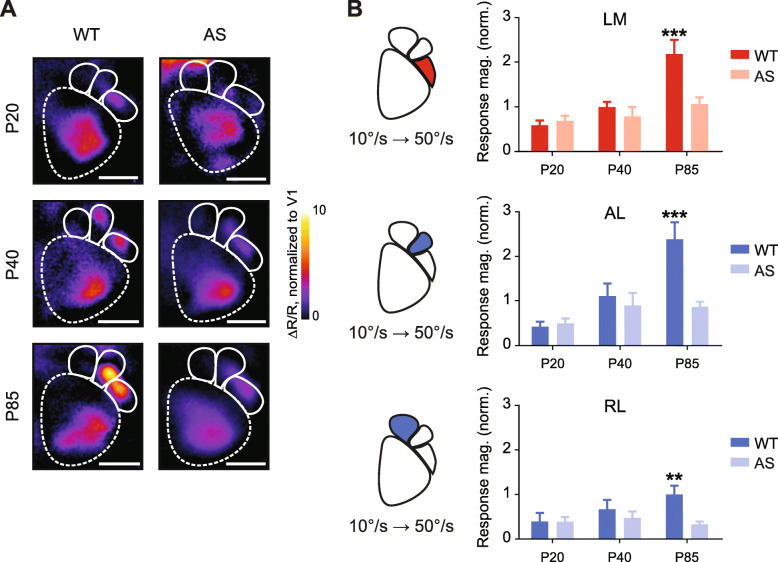
Enhanced HVA response to slow-fast gratings emerges late in development in WT but not AS mice. **a** Representative magnitude maps of primary and HVA responses to slow-fast gratings at P20, P40, and P85. ΔR/R, normalized to V1. Scale bar, 1 mm. **b** Quantification of responses to slow-fast gratings in LM, AL, and RL of WT and AS mice. LM: age effect (*p* = 0.0007), genotype effect (*p* = 0.0473), interaction effect (*p* = 0.0449). AL: age effect (*p* = 0.0014), genotype effect (*p* = 0.0283), and interaction effect (*p* = 0.0242). RL: genotype effect (*p* = 0.0427). Two-way ANOVA. Bonferroni post hoc. **p < 0.01; ***p < 0.001

We sought to investigate the deficit in more detail by varying the starting stimulus speed. We measured responses to four different starting speeds: 0°/s, 5°/s, 7.5°/s, and 10°/s (in all cases, periodically speeding up to 50°/s). We then generated starting-speed response curves for each HVA in each mouse (Fig. 1d). Areas LM, AL, and RL in WT mice showed steeper starting-speed response curves than AS mice (two-way ANOVA; LM: stimulus *F*_(3,80)_ = 7.898, *p* = 0.0001, genotype *F*_(1,80)_ = 15.45, *p* = 0.0002, interaction *F*_(3,80)_ = 3.424, *p* = 0.0211; AL: stimulus *F*_(3,80)_ = 15.54, *p* < 0.0001, genotype *F*_(1,80)_ = 29.76, *p* < 0.0001, interaction *F*_(3,80)_ = 6.290, *p* = 0.0007; RL: stimulus *F*_(3,80)_ = 14.93, *p* < 0.0001, genotype *F*_(1,80)_ = 14.48, *p* = 0.0003, interaction *F*_(3,80)_ = 6.188, *p* = 0.0008). Areas LM, AL, and RL increased activity modulation in response to slow-fast gratings in WT mice (Tukey’s multiple comparisons; WT LM: 10°/s vs 0°/s *p* < 0.0001, 10°/s vs 5°/s *p* < 0.0001, 10°/s vs 7.5°/s *p* = 0.0023; WT AL: 10°/s vs 0°/s *p* < 0.0001, 10°/s vs 5°/s *p* < 0.0001, 10°/s vs 7.5°/s *p* < 0.0001; WT RL: 10°/s vs 0°/s *p* < 0.0001, 10°/s vs 5°/s *p* < 0.0001, 10°/s vs 7.5°/s *p* < 0.0001), while those in AS mice remained unchanged across all four stimuli (Fig. 2c). At the 10°/s→50°/s (slow-fast grating stimulus) point of the speed response curve, modulation is reliably observed in WT mice, but the visually driven signal in AS mice is approximately half that in WT mice. These results indicate that the slow-fast grating stimuli modulated activity in HVA circuitry in WT mice, but not as much so in AS mice, particularly when the starting speed is higher.

### HVA responses to slow-fast stimuli develop after P40

We sought to determine when the HVA response to the slow-fast stimulus developed. We examined two developmental time points—postnatal days 20 (P20) and 40 (P40)—to assess the development of HVA responses [6, 17, 37, 43], both before and after a critical period for visual cortical development in mice [35]. We observed that the slow-fast grating stimulus evoked weak cortical activity in HVAs of both P20 and P40 mice (Fig. 2a). In area LM, we found significant effects of age (*F*_(2,39)_ = 8.733, *p* = 0.007) and genotype (*F*_(1,39)_ = 4.197, *p* = 0.0473) as well as an interaction effect (*F*_(2,39)_ = 3.364, *p* = 0.0449) (two-way ANOVA; Fig. 2b). There was a significant difference between WT and AS mice at P85 but not at younger ages (*p* < 0.001, Bonferroni’s, Fig. 2b). In AL, we also found significant effects of age (*F*_(2,39)_ = 7.823, *p* = 0.0014) and genotype (*F*_(1,39)_ = 5.188, *p* = 0.0283), as well as an interaction effect (*F*_(2,39)_ = 4.102, *p* = 0.0242)(two-way ANOVA, Fig. 2b). Finally, RL only showed the effect of genotype (*F*_(1,39)_ = 4.391, *p* = 0.0427), with a significant difference between WT and AS mice at P85 (*p* < 0.01, Bonferroni’s, Fig. 2b). Thus, the response to slow-fast gratings in HVAs of WT mice develops between P40 and P85, after the critical period for ocular dominance plasticity.

### Genotypic differences in stimulus-evoked activity at the cellular level

Measurements obtained with ISOI provide a readout of neuronal activity in response to stimuli [36, 44], but responses at the single-cell level are not resolved. *Ube3a* is expressed in both excitatory neurons and inhibitory interneurons in the cortex [20, 22, 45], where it regulates synaptic development and function in both cell classes [20, 22, 23]. Accordingly, the stimulus-specific effect observed with ISOI could arise from altered function in either excitatory neurons, inhibitory interneurons, or both populations of cells. To distinguish among these possibilities, we turned to two-photon calcium imaging and recent advances in genetically encoded calcium indicators [46, 47]. We generated AS model mice and their WT littermates that express the genetically encoded calcium indicator GCaMP6s under the control of either the Nex promotor (to drive expression in excitatory neurons) or the Gad2 promotor (to drive expression in inhibitory interneurons). We used two-photon population calcium imaging to measure visually evoked responses of individual neurons in these genetically engineered mouse lines.

**Fig. 3:**
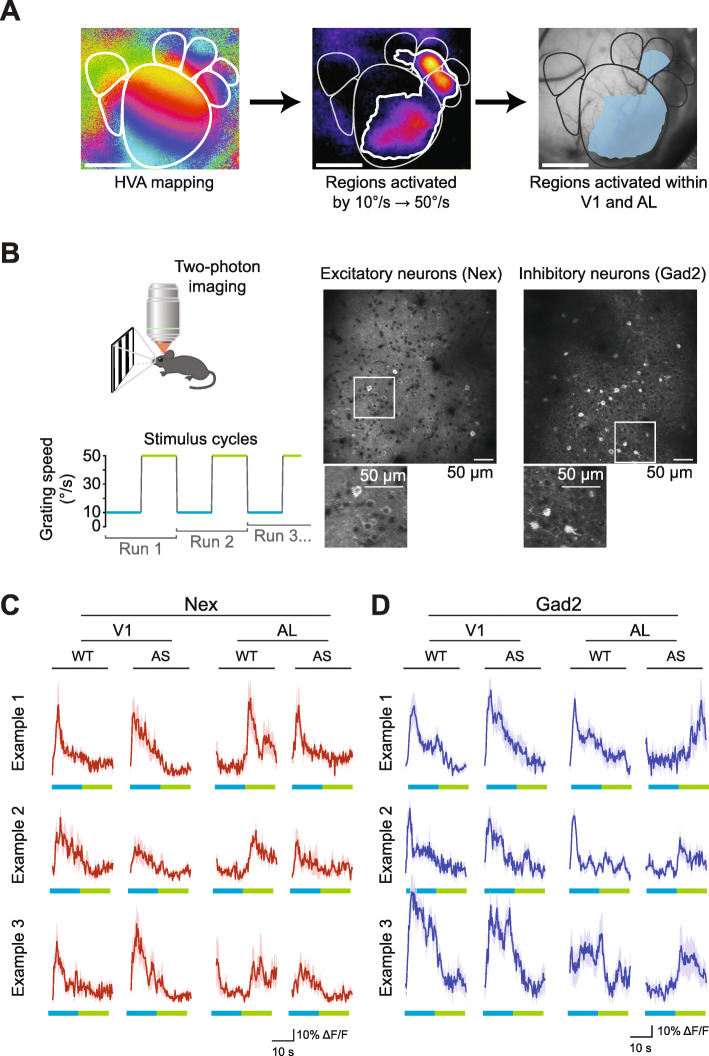
Two-photon population calcium imaging of stimulus-evoked responses in inhibitory and excitatory neurons. **a** ISOI is used to identify V1 and HVA boundaries as well as cortical areas that are responsive to the slow-fast gratings to create two sets of ROIs. Areas activated within V1 and AL were registered with respect to the vasculature. Scale bar, 1 mm. **b** Schematic of two-photon imaging setup. Stimuli are presented to the contralateral (left) eye via an optically isolated monitor. The vasculature map was used to target activated areas within V1 and AL. Sample FOV projection from V1 of a *NexCre/+::Ai96Ds/+::Ube3a*^*m+/p+*^ mouse (Nex, excitatory neurons) and a *Gad2Cre/+::Ai96Ds/+::Ube3a*^*m+/p*+^ mouse (Gad2, inhibitory interneurons). **c** Sample traces from excitatory neurons in V1 and AL of both WT and AS mice. Mean is plotted with SEM. **d** Sample traces from inhibitory interneurons in V1 and AL of both WT and AS mice. Mean is plotted with SEM

We chose to focus on AL, the area that the ISOI experiments indicated was most affected in the AS model. To identify appropriate cortical areas for imaging, we first performed ISOI to identify the locations of HVAs as well as the subregions within these areas that respond to the slow-fast stimulus (Fig. 3a). These ROIs were registered to the vessel map of each mouse to identify activated areas within V1 and AL (Fig. 3a). Mice were then transferred to a two-photon microscope for imaging (Fig. 3b). We obtained strong cellular responses in both excitatory neurons and inhibitory interneurons (regardless of the genotype or visual area) in response to a slow-fast grating stimulus (Fig. 3c and d). Although there was no statistical difference in the peak amplitudes of calcium transients between WT and AS groups (*p* > 0.2), there was a marked difference in the temporal phase in which the peak transients occurred relative to the stimulus cycle (in both Nex-GCaMP6s and Gad2-GCaMP6s mice) (Fig. 3c and d). Due to the periodicity of the stimulus, two transition points occurred that could drive activity modulation: the “speed up” (SU) from 10°/s to 50°/s or the “slow down” (SD) from 50°/s to 10°/s. To quantify the stimulus-response phase relationships, we analyzed the stimulus-evoked response by breaking the data into these two epochs.

### Genotypic differences in responses of excitatory neurons

**Fig. 4:**
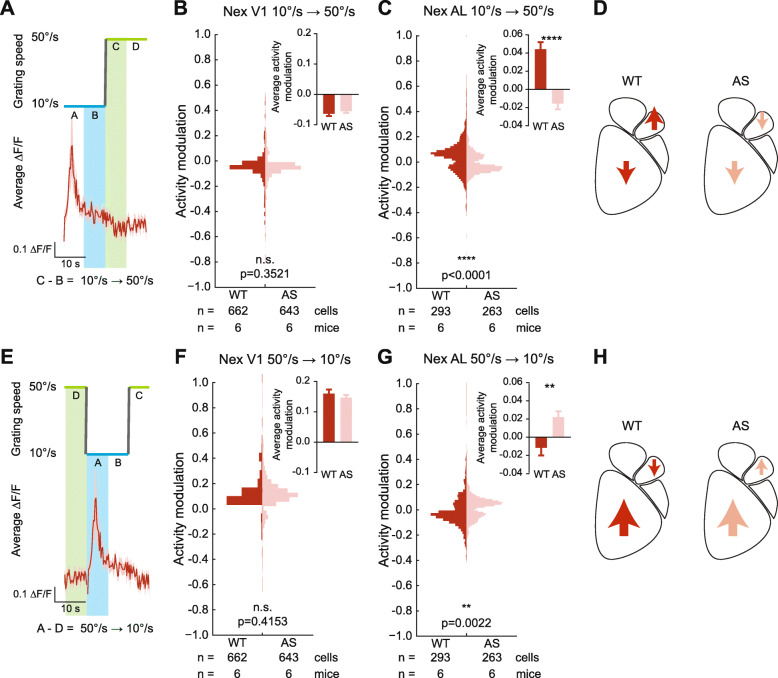
WT and AS mice show opposite patterns of modulation of excitatory neurons in AL in response to speed up and slow down stimulus components. **a** Diagram of how activity modulation in response to “speed up” (SU) was calculated. The difference in average activity before and after the 10➔50°/s transition was calculated for each cell responsive to the stimulus. **b**, **c** Population responses of V1 (**b**) and AL (**c**) excitatory neurons in response to the SU component. Insets show average activity modulation across the cell population. **d** Summary of average excitatory activity modulation to SU by region and genotype. **e** Diagram of how activity modulation in response to “slow down” (SD) component was calculated. The difference in average activity before and after the 50➔10°/s transition was calculated for each cell response to the stimulus. **f**, **g** Population responses of V1 (**f**) and AL (**g**) excitatory neurons in response to the SD component. Insets show average activity modulation across the cell population. **h** Summary of average excitatory activity modulation to SD by region and genotype. Two-tailed *t* test. **p < 0.01; ****p < 0.0001

We calculated the neuronal responses to the SU epoch by taking the difference in average activity before and after the 10➔50°/s transition (Fig. 4a). In excitatory layer 2/3 neurons, we found no genotypic difference between neuronal responses in V1 during SU (*p* = 0.3521, Fig. 4b). In both WT and AS mice, excitatory neuron activity in V1 decreased during SU (−0.063 ± 0.007 and −0.055 ± 0.006 respectively). This decrease was due to a return to baseline after a response to the SD. In AL, however, the SU transition produced a bimodal distribution of cellular responses in both WT and AS mice. The distribution pattern was inverted between the two genotypes, with a net increase of excitatory responses in AL of WT mice (0.044 ± 0.007) and a net decrease in AL of AS mice (−0.016 ± 0.006) (*t*_(554)_ = 5.895, *p* < 0.0001) (Fig. 4c). Thus, the SU transition results in a similar excitatory activity pattern in V1 of WT and AS mice (Fig. 4d), but opposite patterns of activity in AL, with WT mice showing net increases and AS mice showing net decreases in excitatory neuron activity (Fig. 4d).

The response to the SD transition of the stimulus was also calculated, by taking the difference in average activity before and after the 50➔10°/s transition (Fig. 4e). As with the SU transition, there was no genotypic difference in V1 activity (*p* = 0.42, Fig. 4f). Excitatory neurons in V1 of both genotypes increased activity in response to SD (0.16 ± 0.013 and 0.15 ± 0.009 respectively). In AL, the SD component produced a bimodal population response, similar to the response to SU in both WT and AS mice. The average activity modulation was a net increase in excitatory activity in AS mice (0.022 ± 0.007), and a slight net decrease in excitatory activity in WT mice (−0.012 ± 0.009, *t*_(554)_ = 3.077, *p* = 0.0022) (Fig. 4g). In summary, the SD component produced an increase in excitatory neuron activity in V1, regardless of genotype, as well as an increase in AL of AS mice not seen in WT AL (Fig. 4h).

### Genotypic differences in responses of inhibitory interneurons

**Fig. 5:**
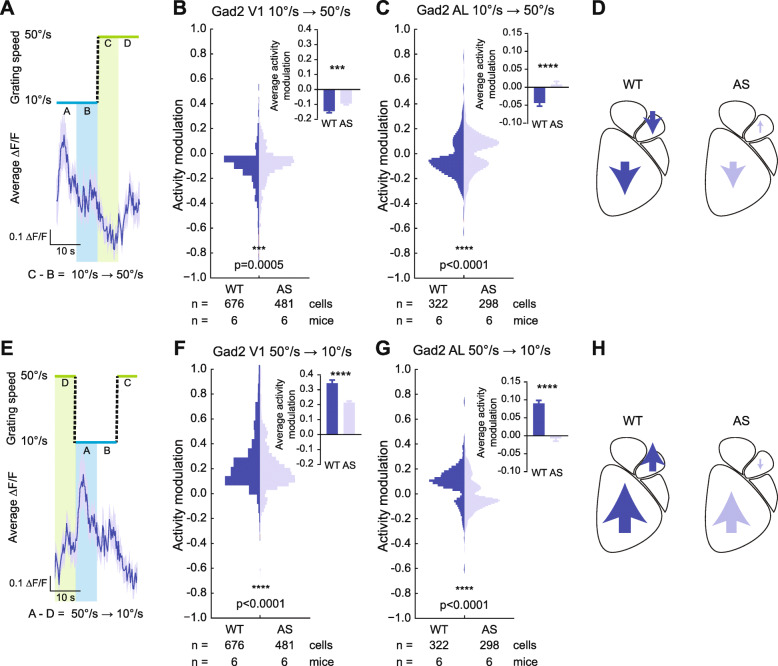
Stimulus-evoked inhibitory activity modulation is reduced in AS mice. **a** Diagram of how activity modulation in response to “speed up” (SU) was calculated. The difference in average activity before and after the 10➔50°/s transition was calculated for each cell responsive to the stimulus. **b**, **c** Population responses of V1 (**b**) and AL (**c**) inhibitory neurons in response to the SU component. Insets show average activity modulation across the cell population. **d** Summary of average inhibitory activity modulation to SU by region and genotype. **e** Diagram of how activity modulation in response to “slow down” (SD) component was calculated. The difference in average activity before and after the 50➔10°/s transition was calculated for each cell response to the stimulus. **f**, **g** Population responses of V1 (**f**) and AL (**g**) inhibitory neurons in response to the SD component. Insets show average activity modulation across the cell population. **h** Summary of average inhibitory activity modulation to SD by region and genotype. Two-tailed *t* test. ***p < 0.001; ****p < 0.0001

Next, we measured responses of inhibitory interneurons to the slow-fast stimulus. We calculated activity modulation to the SU transition as before (Fig. 5a). The SU transition decreased interneuron activity in V1 in both WT (−0.14 ± 0.01) and AS (−0.095 ± 0.008) mice, with WT mice showing greater decreases in activity (*t*_(1155)_ = 3.505, *p* = 0.0005) (Fig. 5b). In AL, the SU component produced opposite activity patterns by genotype, with the WT population on average decreasing in activity (−0.044 ± 0.009) and the AS population showing a slight increase on average (0.0073 ± 0.009, *t*_(618)_ = 4.081, *p* < 0.0001) (Fig. 5c, d).

Responses to the SD component of the stimulus were calculated by taking the average difference before and after the 50➔10°/s transition (Fig. 5e). During the SD transition, both WT (0.34 ± 0.02) and AS (0.21 ± 0.01) mice showed a strong increase in inhibitory activity in V1 (*t*_(1155)_ = 4.877, *p* < 0.0001) (Fig. 5f). In AL, however, WT interneurons showed a strong increase in activity to the SD transition (0.09 ± 0.008), while AS interneurons showed a slight net decrease in average stimulus evoked-activity (−0.0073 ± 0.008, *t*_(618)_ = 8.614, *p* < 0.0001) (Fig. 5g, h). Taken together, the increases in WT inhibitory activity in response to the SD and the decreases at the SU phase suggest that in WT mice, inhibitory activity was locked to the stimulus (Fig. 5h). In contrast, AS mice showed weak inhibitory interneuron activity in AL throughout the stimulus cycle (Fig. 5h).

### Genotypic differences in aggregate neuronal populations

The combined findings from excitatory and inhibitory neurons offer a potential cellular level explanation of the deficits measured with ISOI. We pooled the activity modulation results for both excitatory (Nex^Cre/+^ experiments) and inhibitory (Gad2^Cre/+^ experiments) neurons by genotype to examine aggregate stimulus-evoked activity modulation. During the SU epoch of the stimulus, in V1 of both WT and AS mice, a decrease in inhibitory activity dominates, with WT mice showing a larger decrease in inhibitory response (WT −0.11 ± 0.007, AS −0.072 ± 0.004, *t*_(2460)_ = 3.883, *p* = 0.0001). During the same epoch, in AL of both WT and AS mice, the activity modulations of excitatory and inhibitory neurons largely canceled each other out (in WT −0.002 ± 0.006; in AS −0.0034 ± 0.006; no genotypic difference, *p* = 0.8601). Our results indicate that the aggregate response in both genotypes during the SU epoch was a decrease in neuronal activity in V1 and a net lack of activity modulation in AL.

During the SD epoch, however, we observed differential genotype-dependent responses. An increase in inhibitory interneuron activity in both V1 and AL of WT mice outweighed the decrease in excitatory neuron activity during the same stimulus epoch to produce a net increase in neuronal activity (V1: *t*_(2460)_ = 5.027, *p* < 0.0001, WT: 0.25 ± 0.01, AS: 0.18 ± 0.007; AL: *t*_(1174)_ = 4.296, *p* < 0.0001, WT: 0.042 ± 0.006, AS: 0.0064 ± 0.005). These results suggest that the activity modulation observed with ISOI in AL of WT mice arises from increases in inhibitory interneuron activity during the SD epoch of the stimulus, and the absence of activity modulation in AL of AS mice is due to a lack of inhibitory interneuron activity surge during the SD epoch of the stimulus.

## Discussion

We used in vivo ISOI and 2-photon imaging to reveal a functional deficit in HVAs caused by the lack of maternal *Ube3a* expression. HVAs of WT mice exhibited strong activity modulations in response to a grating stimulus that varies in drift speed [40]. In contrast, HVAs in AS mice exhibited only weak activity modulation in response to the same stimulus. We found that this deficit in AS mice emerges after P40, relatively late in the development of visual circuitry [37, 48]. Further, we identified that a decrease in stimulus-evoked interneuron activity (and, to a lesser extent, excitatory neuron activity) underlies this deficit in HVAs of AS mice.

A lack of maternal *Ube3a* expression causes widespread synaptic and cellular deficits, including abnormalities in V1 microcircuits, impairments in ocular dominance plasticity, and deficits in experience-driven dendritic spine maintenance [20–23, 49]. Given these deficits, a visual cortical function could have been expected to be broadly dysfunctional. However, we found that visually evoked activity in V1 and HVAs of AS mice was largely normal, at least for the still-fast grating stimulus and the RDK stimulus [50]. In contrast, the slow-fast grating stimulus modulated activity in HVAs more in WT mice [40] than in AS mice. Further, the genotypic differences identified by the speed response curve imply that the abnormalities in HVA activity could represent a stimulus threshold deficit. Since the deficit is more pronounced for smaller speed changes, this suggests a role for UBE3A in shaping circuitry for fine aspects of visual processing.

The decrease in HVA activity modulation appears to be mostly due to decreases in interneuron activity. While AS mice did have differences in activity modulation in both excitatory neurons and inhibitory interneurons, we found larger abnormalities in interneuron activity, with the cell population exhibiting weak modulation of activity regardless of the phase of the stimulus. This finding is consistent with the observation that the loss of maternal *Ube3a* produces deficits in presynaptic vesicle cycling and release specifically in interneurons, associated with a decreased inhibitory drive onto excitatory neurons [22]. Reduced responses in inhibitory interneuron activity have also been observed in a mouse model of Rett syndrome [51], and this suggests similarities of mechanisms across two neurodevelopmental disorders. We have previously performed in vitro studies to examine differences between global and interneuron-specific deletion of Ube3a [20], which revealed both cell-autonomous and non-autonomous electrophysiological phenotypes.

While speculative, the larger deficits in stimulus-evoked inhibitory activity we observed could be due to this presynaptic accumulation of vesicles in interneurons, impairing effective inhibitory signaling that is crucial for regulating the timing of neuronal activity in cortical circuits [52–54]. This would also explain the abnormal stimulus-evoked activity patterns in excitatory neurons that we observed in AL of AS mice: impaired interneuron activity would be unable to effectively regulate excitatory neurons, leading to disorganized excitatory activity that does not effectively represent to the stimulus. This idea is compatible with the finding that a loss of maternal *Ube3a* in interneurons is sufficient to produce pathology, specifically increasing seizure susceptibility and EEG delta power [20]. Thus, the lack of a uniform stimulus-evoked response in interneurons could underlie the lack of stimulus-evoked activity modulation in HVAs of AS mice.

To examine how the deficits develop, we examined mice at ages around the critical period for ocular dominance plasticity [35, 48]. The HVA response in WT mice to the slow-fast stimulus strengthened after P40, but in AS mice, the response changed little from P20 to adulthood. Loss of *Ube3a* produces abnormalities in experience-dependent refinement of cortical circuitry [21, 23, 49] and several inhibitory deficits in AS mice emerge only with development [22]. Based on the calcium imaging results, we would expect to see a reduced inhibitory drive to pyramidal neurons in HVAs, as reported for V1 [22], and a lower excitatory synaptic drive for inhibitory interneurons. The development of appropriate visual cortical responses to fine stimulus distinctions appears to be dependent on intact *Ube3a*. Visually evoked responses in V1 are similar in many metrics between AS and WT mice [50]. V1 develops earlier than HVAs [37]**,** and as a consequence might not have the same deficits in activity-dependent refinement of circuitry compared with later-developing HVAs. These findings also imply that there may be a developmental window wherein the circuitry is amenable to therapeutic intervention or other manipulations.

What might the functional consequences be of the observed deficits in HVAs? In humans, sensitivity to speed perception emerges relatively late in development, with maturation continuing even at 16 years of age [55, 56]. While different speeds and task configurations were used for these human experiments, our finding that the cortical response to a low saliency speed-change stimulus develops relatively late in WT mice is in line with these human findings. It is possible that a developmental mechanism that fine-tunes the visual system to subtler aspects of visual perception, especially speed discrimination, is conserved across species and involves UBE3A and properly functioning interneurons. Testing this experimentally, with visually driven behavior, could be challenging due to a known motor and learning deficits that also arise with a lack of maternal *Ube3a* [16, 17, 57]. However, touchscreen-based tests could be successful in assessing visually driven behavior [32, 34, 58].

Together, these data show that higher sensory processing might be generally intact except when challenged by more subtle visual discriminations. Based on this, we argue that the in vivo consequences of abnormalities caused by the loss of *Ube3a* in interneurons produce circuitry that is grossly normal, except when strained by subtle stimuli. Thus, changes to stimulus saliency bring out genotypic differences in cortical circuitry lacking maternal *Ube3a*.

## Conclusions

We identified a neural response deficit in higher visual processing in a mouse model of Angelman syndrome (AS). The deficit manifests as a weak response to drifting edges that change in speed. It emerges developmentally, and it is a larger deficit in higher visual areas (HVAs) compared with the primary visual cortex. We used two-photon imaging to examine the cell-type basis for the deficit and identified a weak response of inhibitory interneurons in an HVA. These results support a model in which lower, primary sensory areas exhibit relatively weak deficits, yet these deficits impair the development of higher cortical areas and ultimately lead to larger deficits of neural circuitry that underlie behavior.

## Materials and methods

### Animals

All animal procedures were approved by the Institutional Animal Care and Use Committee of the University of North Carolina School of Medicine and were performed in accordance with the guidelines of the U.S. National Institutes of Health. AS mice (*Ube3a*^*m–/p+*^) [18] (Jackson Labs Stock # 016590; C57BL6) and their wildtype littermates were generated by breeding *Ube3a*^*m+/p–*^ females with wildtype males. Mice for P20 ISOI experiments were removed from the nest and immediately used for imaging. Mice for P40 ISOI, P85 ISOI, and two-photon experiments were weaned into cages of 2–4 mice and housed until used for experiments. Mice for two-photon experiments were generated using the Ai96 mouse line (B6;129S6-Gt(ROSA)26Sor^tm96(CAG-GCaMP6s)Hze^/J; Jackson Labs Stock # 024106), which conditionally expresses GCaMP6s under the control of a floxed-STOP cassette. To examine excitatory cell activity, double transgenic—*Nex*^*Cre/+*^ *:: Ai96/Ai96* or *Gad2*^*Cre/+*^ *::Ai96/Ai96* [59]— males were generated and were bred with *Ube3a*^*m+/p–*^ females to produce triple-transgenic experimental mice: *Nex*^*Cre/+*^ *:: Ai96/+ :: Ube3a*^*m–/p+*^ or *Nex*^*Cre/+*^ *:: Ai96/+ :: Ube3a*^*m+/p+*^. To examine interneuron activity, double transgenic—*Gad2*^*Cre/+*^ *::Ai96/Ai96* (Jackson Labs Stock # 010802; C57BL6)*—*males were generated and were bred with *Ube3a*^*m+/p–*^ females to produce triple-transgenic experimental mice: *Gad2*^*Cre/+*^ *:: Ai96/+ :: Ube3a*^*m–/p+*^ or *Gad2*^*Cre/+*^ *:: Ai96/+ :: Ube3a*^*m+/p+*^. Mice of both sexes were used. Mice were raised in a temperature- and humidity-controlled room on a 12-h light/dark cycle and provided ad libitum access to food and water. All experiments were conducted during the animals’ light cycle.

### Surgical procedures

Anesthesia was induced with 5% isoflurane that was reduced to 1.0–2.5% isoflurane for surgery. After the initial induction of anesthesia, 2.5 mg/kg chlorprothixene was administered (i.p.). Ophthalmic ointment (Lacri-lube, Allergan) was applied to the eyes prior to surgery and removed immediately prior to imaging. Throughout the surgery, body temperature was maintained via a heating pad. The scalp was resected over the right visual cortex and a 4-mm craniotomy performed, exposing the cortex. For ISOI experiments, physiological saline was added to cover the cranial window and mice were then transferred to the ISOI rig. For calcium imaging experiments, a 1-mm glass coverslip was placed over the craniotomy and saline added before transferring the mouse to the ISOI rig.

### ISOI imaging and visual stimuli

All imaging was performed blind to genotype. Intrinsic signal optical imaging (ISOI) was used to identify and measure cortical activity in V1 and the HVAs of mice [36]. Mice were maintained on 0.5% isoflurane for the duration of imaging. The brain was illuminated with 700-nm light and imaged with a tandem lens macroscope focused 600 μm into the brain from the vasculature. Images were acquired at 30 Hz with a 12-bit CCD camera (Dalsa 1 M30), frame grabber and custom LabView software (David Ferster, Northwestern University, with in-house modifications by Jeffrey Stirman). These 12-bit images were binned in software four times temporally and 2 × 2 spatially, resulting in images with 16-bit pixel data. From these binned images, Fourier analysis of each pixel’s time course was used to extract the magnitude and phase of signal modulation at the stimulus frequency. This produced phase maps of the cortical response, used to map retinotopy, as well as magnitude maps of cortical areas modulated by the stimulus, used to measure the strength of the visually evoked response.

Visual stimuli were presented using Psych ToolBox to the contralateral eye relative to the imaged hemisphere via a Dell LCD monitor (Dell U2711b, 2560 × 1440 pixels, 60 Hz). Stimulus frames were modified to correct for the flat surface of the monitor (http://labrigger.com/blog/2012/03/06/mouse-visual-stim/). Retinotopy was mapped by showing the animal a single bar drifting across the screen to identify V1 and higher visual areas (horizontally for azimuth and vertically for elevation). Mice were then shown the experimental stimulus set and the magnitude of activity for each ROI quantified.

Still-fast gratings: This stimulus consisted of a 50° patch in the center of the visual field, displaying square-wave generated black and white bars (0.04 cycles/°) that changed in drift velocity from 0°/s (6 s) to 50°/s (2 s). This stimulus was presented for 50 8-s cycles.

Slow-fast gratings: This stimulus consisted of a 50° patch in the center of the visual field, displaying square-wave generated black and white bars (0.04 cycles/°) that changed in drift velocity from 10°/s (6 s) to 50°/s (2 s). The change was instantaneous. This stimulus was presented for 50 8-s cycles.

Other grating stimuli: The drift velocity parameter was explored further by changing the slower speed, with 5°/s to 50°/s and 7.5°/s to 50°/s being used. In all other regards, these two stimuli were identical to still-fast and slow-fast gratings.

Random-dot kinematogram (RDK): A random-dot kinematogram with 0% coherence was also used. This stimulus was presented as a patch in the center of the visual field with dots moving randomly for 6 s and remaining stationary for 2 s.

### Two-photon imaging and visual stimuli

All imaging was performed blind to genotype. Using ISOI as described above, V1 and HVA boundaries were identified as well as the boundaries of cortical areas that were visually responsive to slow-fast gratings (Fig. 3a). These two sets of ROIs were registered with respect to the vasculature and all areas targeted for imaging were chosen relative to vascular landmarks within areas activated by the slow-fast gratings within V1 and AL. The mouse was immediately transferred to a Zeiss 7MP controlled by Zen10 software for two-photon imaging and maintained on 0.25–0.5% isoflurane for the duration of the experiment. Fields of view were identified based on vascular landmarks using widefield microscopy, illuminated by an HBO lamp. Imaging was then switched to two-photon microscopy, with the laser at 950–980 nm. Laser power varied between 17 and 40% (of approx. 1140 mW) for *Nex*^*Cre/+*^ experiments or 20–45% for *Gad2*^*Cre/+*^ experiments. Visually evoked calcium transients reported by GCaMP6s were recorded in mice at P85. Data were acquired as a time-series at 267.27 ms per frame, with 0 s between frames, 449.9 μm × 452 μm with a pixel size of 1.3 μm. Imaging was performed in layer 2/3, with multiple depths within each field of view acquired if possible.

Stimuli were displayed to the contralateral eye relative to the imaged hemisphere at 30 Hz via an optically isolated monitor (Digital TFT LCD, 480 × 272 pixels) controlled by custom LabVIEW software. The monitor shroud ensured a consistent distance (60 mm) between the eye of the mouse and the center of the screen as well as a consistent view angle (46°, 0.1338°/pixel) (Fig. 3b). As a result, the visual stimuli presented via this monitor were analogous to the 50° patch stimuli used with ISOI. Three stimuli were used in each field of view, with each stimulus triggered by the frame synchronization output from the Zeiss 7MP which was monitored by a NI DAQ Board (NI USB-6501).

Gratings with multiple orientations: In order to not bias our field of view selection, an orientation grating stimulus was used to confirm regions of visually responsive cells. This stimulus consisted of two epochs: a gray screen displayed for 30 imaging frames and a grating epoch displayed for 40 imaging frames (8 cycling orientations, 5 imaging frames each). This was repeated for 11 cycles.

Still-fast gratings: This stimulus consisted of square-wave generated black and white bars (0.04 cycles/°) that changed in drift velocity from 0°/s (50 imaging frames) to 50°/s (50 imaging frames). This stimulus was presented for 21 cycles.

Slow-fast gratings: This stimulus consisted of square-wave generated black and white bars (0.04 cycles/°) that changed in drift velocity from 10°/s (50 imaging frames) to 50°/s (50 imaging frames). This stimulus was presented for 21 cycles.

### Data quantification

All retinotopic map generation and data quantification were performed blind to genotype and, for developmental studies, age. Retinotopy maps were generated by drawing regions of interest (ROIs) offline in FIJI (Fiji is Just ImageJ; Fiji.sc/Fiji) using the magnitude and phase maps generated by the elevation and azimuth stimuli. The borders of visual areas were identified by reversals (meridians) in the progression of retinotopy [27, 28, 36, 37, 43]. V1, LM, AL, and RL were clearly identified in all mice examined. However, LI, AM, and PM were not consistently delineated by strong phase reversals, so these regions were excluded from further analysis. Once the borders of the HVAs were identified, these boundaries were used to measure the visual activity evoked in each region by the stimulus set.

To quantify the response amplitude of visually evoked activity, we analyzed the magnitude maps as follows. First, we created a duplicate copy of the magnitude map and applied a Gaussian filter (kernel size: 5 × 5 pixels). This filtered map was then processed by applying a threshold to include only a visually responsive cortex and converted into a mask, excluding non-responsive cortical regions. This mask was applied to the original magnitude map generated by the stimulus such that only visually responsive cortex was included in subsequent analysis. The previously defined boundaries of V1 and the HVAs were then applied to the masked magnitude map to obtain average activity measurements across each region in response to the stimulus set. The average activation for each of these HVAs was then normalized to the average activity level of V1 for each animal. This normalization decreases the mouse-to-mouse variability in overall cortical activation and hemodynamic responses and facilitates a comparison of HVA activation within individual animals. A small difference in raw V1 amplitude was observed between AS and WT mice in response to still-fast gratings, *t*(20) = 2.592, *p* = 0.017, but not slow-fast gratings, *t*(20) = 1.738, *p* = 0.098 (Fig. 1, Supp. 2).

To quantify cellular activity recorded with two-photon imaging, each data stack for a field of view was loaded into FIJI. An experimenter blind to genotype then manually identified regions of interest (neuron locations in the data stack) for each field of view and saved these ROIs as a binary mask. Delta F/F was calculated for each ROI after removing the first stimulus presentation (i.e., including the last 20 stimulus presentations) for both still-fast and slow-fast gratings. Cells responsive to slow-fast gratings were determined using an ANOVA comparing the average delta F/F between the two imaging epochs (10°/s and 50°/s) across 20 stimulus presentations. These cells were then included in the analysis of activity modulation. Activity modulation to the “speed up” component of the stimulus was calculated as the difference in average activity before and after the transition from 10°/s to 50°/s in 25 frame bins. Activity modulation to the “slow down” component of the stimulus was calculated as the difference in average activity before and after the transition from 50°/s to 10°/s in 25 frame bins. Both calculations were performed for each cell deemed responsive to slow-fast gratings, providing an analysis of population response to this stimulus.

### Statistical analysis

All data collection, image analysis, HVA identification, and quantifications were carried out blind to genotype, and in the developmental experiments, age. GraphPad Prism 7 was used to calculate two-tailed *t* tests and two-way ANOVAs. Paired, two-tailed *t* tests were used to calculate population differences in Fig. 1c. Tukey’s and Bonferroni post hoc analyses were conducted where noted. Population data are reported in the text as mean ± SEM. All graphs are depicted as mean ± SEM.

## Supplementary information

**Additional file 1.** Supplementary Figure 1: Example retinotopy maps for WT and AS mice. (A) Sample elevation and azimuth maps from 3 WT mice used in the experiments in Figure 1. White lines: ROI boundaries between cortical visual areas. AM and PM are not separated in mouse #3 due to lack of clear boundary. Scale bar, 1 mm. (B) Sample elevation and azimuth maps from 3 AS mice used in the experiments in Figure 1. White lines: ROI boundaries between cortical visual areas. LI not identified in mouse #4. AM and PM are not separated in mice #5 & #6 due to lack of clear boundaries. Scale bar, 1 mm. Regions V1, LM, AL, and RL were consistently identified with high fidelity, regardless of genotype.

**Additional file 2.** Supplementary Figure 2: Raw V1 activity in AS and WT mice in response to two grating stimuli. Schematic of drifting grating stimulus presented as a 50° patch. 0➔50 °/s (still-fast) stimulus produces a statistically significant difference between WT and AS mice. 10➔50 °/s (slow-fast) stimulus produces no genotypic difference. Two-tailed t-test. *, p < 0.05.

**Additional file 3.** Supplementary Figure 3: HVA response to RDK stimulus. Schematic of random dot kinematogram stimulus, presented at 0% coherence. No difference between LM and RL was observed. The RDK stimulus produced a genotypic difference in AL. Two-tailed t-test. *, p < 0.05.

## Data Availability

The data sets generated in the current study are available from the corresponding author on request.
